# Be legally wise: When is parental consent required for adolescents’ access to pre-exposure prophylaxis (PrEP)?

**DOI:** 10.4102/sajhivmed.v21i1.1129

**Published:** 2020-11-10

**Authors:** Ann Strode, Catherine M. Slack, Zaynab Essack, Jacintha D. Toohey, Linda-Gail Bekker

**Affiliations:** 1 School of Law, College and Law and Management Sciences, University of KwaZulu-Natal, Pietermaritzburg, South Africa; 2 HIV/AIDS Vaccines Ethics Group, School of Applied Human Sciences, College of Humanities, University of KwaZulu-Natal, Pietermaritzburg, South Africa; 3 Center for Community-Based Research, Human and Social Capabilities Division, Human Sciences Research Council, Pietermaritzburg, South Africa; 4 The Desmond Tutu HIV Centre, University of Cape Town, Cape Town, South Africa

**Keywords:** parental consent, self-consent, HIV, prevention, minors’ capacity

## Abstract

**Background:**

South African adolescents (12–17 years) need an array of prevention tools to address their risk of acquiring the life-long, stigmatized condition that is HIV. Prevention tools include pre-exposure prophylaxis (PrEP). However, service providers may not be clear on the instances where self-consent is permissible or when parental consent should be secured.

**Aim:**

To consider the legal norms for minor consent to PrEP using the rules of statutory interpretation.

**Setting:**

Legal and policy framework.

**Results:**

We find that PrEP should be interpreted as a form of ‘medical treatment’; understood broadly so that it falls within the ambit of one of consent norms in the *Children’s Act*. When PrEP is interpreted as ‘medical treatment’, then self-consent to PrEP is permissible for persons over 12 years, if they have the mental capacity and maturity to understand the benefits, risks, social and other implications of the proposed treatment. Currently, PrEP is only licensed for persons over 35 kg. Reaching the age of 12 years is a necessary but not sufficient criteria for self-consent and service-providers must ensure capacity requirements are met before implementing a self-consent approach. Decisional support and adherence support are critical.

**Conclusions:**

We recommend that *service-providers* should take steps to ensure that those persons who meet an age requirement for self-consent, also meet the capacity requirement, and that best practices in this regard be shared. We also recommend that *policy makers* should ensure that PrEP guidelines are updated to reflect the adolescent consent approach articulated above. It is envisaged that these efforts will enable at-risk adolescents to access much needed interventions to reduce their HIV risk.

## Adolescent human immunodeficiency virus risk and pre-exposure prophylaxis

Globally young people are especially vulnerable to human immunodeficiency virus (HIV).^1,2,3,4^ Human immunodeficiency virus prevalence amongst adolescents and young adults in South Africa remains skewed. In 2017, the HIV prevalence amongst females was higher than their male counterparts (5.8% vs. 4.7% amongst 15–19 year olds and 15.6% vs. 4.8% amongst 20–24 year olds).^5^ In the same year, 66 000 new HIV infections occurred amongst adolescent girls and young women in South Africa.^5^ Likewise, young men having sex with men (MSM) in South Africa are highly vulnerable to HIV infection.^5^

There is now good evidence that oral pre-exposure prophylaxis (PrEP) taken daily, as part of a combination prevention package, can protect HIV-negative adults against HIV acquisition.^6,7,8,9^ The US Federal Drug Administration has, based on safety data, licensed oral combination of Tenofovir (TDF)/Emtricitabine (FTC) for HIV prevention for at-risk adolescents with body weights above 35 kg (Bekker, personal communication, 9 Jun 2020). The South African Health Products Regulatory Authority (SAHPRA) has similarly approved a fixed-dose combination of tenofovir disoproxyl fumarate and emtricitabine for PrEP (for adults and adolescents > 35 kg).^7^

In addition to oral PrEP, which is proven and registered for use as HIV prevention, there are additional PrEP options that have different routes of administration and less frequent dosing including long-acting injectable PrEP and vaginal rings. These are at various stages in the development pipeline, with the dapivirine vaginal ring furthest along also currently under review by regulatory agencies. This means adolescents may soon have more choices about the form of PrEP available to them (Bekker, personal communication, 9 Jun 2020).

Providing at-risk populations with access to PrEP is described as a key objective within the South African National Strategic Plan on HIV, tuberculosis (TB) and sexually transmitted infections (STIs): 2017–2022.^9^ Initially, the Department of Health operationalised this objective by targeting sex workers and MSM, but this has now been expanded to include other at-risk populations such as university students and young women.^10^ To date public sector roll-out has lagged, and PrEP is mostly available through demonstration projects, clinical research sites and the private healthcare sector.^11,12^ However, South Africa is now in the process of expanding access, with 3000 facilities being able to provide oral PrEP. Within this community-based approach, self-presenting adolescents who are > 35 kg and are deemed to be at risk of HIV acquisition will be eligible to access oral PrEP.

Although PrEP is registered for use in persons > 35 kg, there is no policy that deals with consent to this product by persons under 18 years. For example, the current South African HIV Clinicians guidelines do not address the consent approach for adolescent access to PrEP. These guidelines are currently being updated, and it is understood that the new version which will be published in November 2020 will include a recommended consent approach for persons under 18 years.^12^ The unintended consequences of this lack of policy on adolescent consent to PrEP is that it is unclear whether adolescents can self-consent or require parental consent for access to PrEP.

In this article, we describe the current legal framework for adolescent consent to health interventions including ‘medical treatment’. We examine whether adolescents can consent independently for PrEP in terms of the current legal framework. We conclude with our position on an appropriate consent strategy and recommend that the Department of Health revise current PrEP policies to provide certainty on this issue.

It should be noted that although this article focusses on adolescent consent to PrEP, it has a broader application. As described here, a key issue in the current legal framework is whether the term ‘medical treatment’ in the *Children’s Act,* 2005^13^ is broad enough to encompass prevention interventions such as vaccines. This has implications for adolescent consent to the human papillomavirus (HPV) vaccine and other non-therapeutic health interventions.

## The public health and human rights imperative to ensure adolescent access to pre-exposure prophylaxis

It is both a human rights and public health imperative to ensure that adolescents have access to tools to minimise their HIV risk.^4^ Access requires an evaluation of barriers, including legal barriers in the form of parental consent requirements.^2^ Research from the United States of America has shown that parental consent may act as a legal barrier to adolescents accessing sexual and reproductive health services.^14^ One study indicated that up to one-fifth of adolescents who were surveyed did not want their parents to be involved in the consent process.^15^ Other studies have shown, for example, that a greater number of adolescents volunteered for services such as HIV testing once they were able to provide independent consent.^15^ Furthermore, many adolescents are deterred from accessing abortion and contraception services by parental consent because they fear parental disappointment, sanction or retaliation.^15^ Similarly, there are concerns that parental consent might impede access to HIV prevention packages for adolescents for similar reasons.^4,16^

## The current legal framework for child consent to health interventions

### Self-consent to specified health interventions

The *Children’s Act* states that full legal capacity is attained at 18 years; however, persons below this age may, in certain circumstances, legally self-consent to a range of specified health services, as we have noted elsewhere.^2,3,16^ Sections 12 and 129–135 of the *Children’s Act*^13^ deal with the consent requirements for medical treatment, surgical operations, HIV testing, male circumcision and contraceptives.^2,3^ The *Children’s Act* refers expressly to three current forms of HIV prevention, namely male circumcision, condoms (under contraceptives) and HIV testing, and sets ages at which adolescents may self-consent to the intervention.

As set out in earlier articles, consent to ‘medical treatment’ is a general category in the Act that covers a range of non-specified health interventions.^13^ Section 129 provides that a child may consent independently to ‘medical treatment’ if they are older than 12 years and they have the ‘mental capacity to understand the benefits, risks, social and other implications’ of the proposed treatment.^2,3^ If a child is below the age of 12 years or lacks capacity, proxy consent must be provided by a parent, guardian or care-giver amongst others.^2,3^

### Self-consent to non-specified health interventions

Whilst the *Children’s Act* provides clarity on consent to most medical interventions for children under 18 years, *it does not directly address the age at which adolescents might self-consent to non-specified preventive interventions* such as PrEP. There are two implications of this lacuna. Firstly, if the *Children’s Act* or any other legislation does not set an age of independent consent to a health service or if the child is below the age specified in law for independent consent, then parental or guardianship consent will be required.^2,3^ Or, secondly, if the intervention is not listed, one could examine any of the other specified health interventions and establish whether they could encompass it. In this instance, the only broad health service that an adolescent can self-consent to is ‘medical treatment’. Thus, one must ask whether something that is not directly therapeutic in nature falls within the ambit of term ‘medical treatment’.

## Establishing the meaning of a statutory term

Where the breadth of a statutory term is unclear, it requires a process of interpretation to establish its scope. There are various approaches to statutory interpretation. Firstly, one can use internal aids such as definitions in the Act. The *Children’s Act* does not contain a definition of ‘medical treatment’ nor does it list a gamut of the therapies that may fall under its umbrella. Furthermore, there is no definition of the term in other legislation.

If we use external aids to statutory interpretation such as a dictionary, there are variations in the way they define ‘medical treatment’. Some recognise medical treatment as an ‘action or manner of treating a patient medically or surgically’.^17^ ‘Medically’ is further defined as ‘a way that relates to medicine’,^18^ And others define the term around the objectives of the treatment, for example ‘the use of drugs, exercises, etc. to improve the condition or an ill, injured person, or to cure disease’.^19^ Neither definition refers expressly to medical treatment including preventing an illness that a healthy person is at risk of contracting.

Where there is limited assistance from internal or external aids the general principles of statutory interpretation must be used. In the Constitutional Court judgement of *Cool Ideas 1186 CC v Hubbard and Another*, the court identified three interconnected elements of statutory interpretation.^20^ Firstly, an examination of the purpose of the provision.^21^ Secondly, a review of its legislative context.^20^ Thirdly, identifying a meaning, which is consistent with the values underlying the Constitution.^20^ The Constitution also provides in section 39 that courts may consider foreign law when interpreting rights.^19^

Firstly, if we apply the principles established in the *Cool Ideas* case, one must establish the purpose of the provision. The term is used within Chapter 7 of the *Children’s Act*, which is headed ‘protective measures relating to health of children’.^13^ As stated here, this section deals largely with consent to a range of health interventions. In the Preamble to the Act, one of its stated purposes is to ‘make provision for structures, services and means for promoting and monitoring the sound physical, psychological, intellectual, emotional and social development of children’ and ‘to promote the protection, development and well-being of children’.^13^ It is submitted that in the light of this discussion, the primary purpose of the consent provisions are to protect children from being treated without informed consent and to ensure their physical well-being is promoted.

Secondly, regarding the context of the provision within the Act - the term is used in a chapter on the protection of the health rights of children.^13^ The historical context of the consent provisions were documented in the *South African Law Reform Commission’s Review of the Child Care Act*: *Final Report*.^21^ This report noted that the previous approach to consent to ‘medical treatment’ served as a barrier to children obtaining appropriate medical care as the age of consent was set at the older age of 14 years and only a limited number of persons could provide proxy consent.^21^ A further contextual issue is that (as we have set out in earlier articles) adolescents are able to consent to various other *specified health prevention interventions*, such as contraceptives and HIV testing.^13^ With regard to both contraceptives and HIV testing, adolescents from the age of 12 are able to access them without parental consent. It is submitted that in this instance the context indicates that the legislator recognised that adolescents did have the capacity to consent to certain preventative health interventions. It would, therefore, be consistent with this approach if medical treatment was interpreted broadly to include other non-specified prevention interventions.

The last consideration from the *Cool Ideas* case is when interpreting a statutory provision one must find an interpretation that is consistent with the constitutional values of human dignity, equality and freedom.^19^ The Constitutional Court has held that the recognition of a child’s dignity requires an acceptance that they have their own, independent and distinctive personalities.^22^ As such, it is argued that a child’s right to inherent dignity requires a recognition of their other rights such as the rights of access to basic healthcare services in section 28 of the Constitution.^19^ A narrow interpretation of the term ‘medical treatment’, which restricts it to therapeutic interventions, would undermine an adolescent’s access to various preventative interventions such as the HPV vaccine or PrEP. This is not consistent with the constitutional value of dignity as it undermines fundamental rights.

Finally, a factor to consider is the approach in foreign jurisdictions. Here, there is limited assistance. A recent review by Taggart et al. found that at present, the only country to explicitly include PrEP as falling within the definition of medical treatment is France.^23^

We submit that based on the interpretation principles described here, it is possible to argue that ‘medical treatment’ ought to be understood broadly as meaning the treatment of a person for a current or *a future condition that they may be at risk of contracting*. Just as, for example, counselling an obese child on the need for a healthier diet and exercise programme could be seen as preventative treatment to reduce their future risk of Type 2 diabetes. We submit that this interpretation is consistent with the purposes and context of the *Children’s Act* and is also consistent with constitutional values.

## Implications of pre-exposure prophylaxis falling within the scope of medical treatment for adolescent consent approaches

Based on the given reasoning, we submit the term ‘medical treatment’ should be interpreted to encompass *interventions to prevent an at-risk person from acquiring a disease*. This means that the term would cover both therapeutic and preventative health interventions. It would also include but not be limited to, for example, the provision of antiretrovirals to prevent HIV acquisition (PrEP). We submit that this is in line with a careful statutory interpretation of the term and it reflects its ordinary practical meaning. As suggested here, many practitioners already provide preventative interventions within the scope of medical treatment such as contraceptive counselling, advice about the HPV vaccine and assistance with healthy diets. In short, this broad interpretation of medical treatment enables doctors to provide more holistic healthcare independently for qualifying adolescents.

With regard to the implication for PrEP being viewed as a form of ‘medical treatment’, there are two requirements for adolescent self-consent. Firstly, they must be ≥ 12 years old, and secondly they must have ‘capacity’. Capacity is the law’s recognition of a person’s ability to perform a juristic act – any action that has legal consequences – such as consenting to medical treatment requires capacity. A person will have capacity if he or she is able to exercise their judgement based on an understanding of the nature and consequences of the decision.^2,5^ In this context the *Children’s Act* provides that a child will have capacity to consent if he or she can understand three elements of the proposed treatment; its ‘benefits, risks, social and other implications’.^2,3,13^

If we apply these factors to consent for PrEP we recommend that in order for an adolescent to self-consent the following criteria should be met, the adolescent would need to be:

- at risk of HIV infection
- weigh more than 35 kg
- 12 years or older
- able to understand the benefits of using PrEP to reduce their risk of HIV, relative to other HIV prevention tools
- mature enough to understand and accept that there are risks attached to using PrEP
- informed that there may be social or other implications associated with taking PrEP such as stigmatisation for being in an ‘at-risk’ category
- able to understand the need for adherence and how this will be integrated into their lives, including the possible need for parental or other support to ensure adherence.

This means that qualifying adolescents will be entitled to privacy regarding their medical treatment choice of HIV prevention.^24^ Given the evolving capacity of adolescents it will be easier for older children to meet these criteria. With younger children, additional decisional supports will need to be put in place to ensure that they are able to exercise sound judgement regarding this form of HIV prevention. If they do not meet these capacity requirements, consent for PrEP will have to be provided by a parent, guardian or caregiver.

Regarding adherence for adolescents, there is not yet robust evidence on effective adherence interventions specifically tailored for adolescents; however, the early demonstration projects have provided some lessons. Access to refills should be as easy as possible, enhanced by regular provider-contact, during and between visits, for example, with a navigator or counsellor.

Support from family and close friends including an intimate partner can be positive, but disclosure of PrEP use has also resulted in social harms such as intimate partner violence. Providers should advise adolescents to seek counselling on safe disclosure.

Short-term incentives to maintain drug levels and plasma drug level feedback have also been studied with varying levels of effectiveness (Bekker, personal communication, 9 Jun 2020). Further implementation research is warranted before this is widely adopted.

## Conclusions and recommendations

South African adolescents need an array of HIV prevention tools to address their risk of acquiring the life-long, stigmatised condition, that is HIV.^1^ This public health crisis requires us to consider current legal norms for consent to prevention tools by adolescents and ensure that service providers *are clear on the instances where self-consent is permissible* or when parental consent should be secured.

We recommend that PrEP should be interpreted as being a form of ‘medical treatment’ so that it falls within the ambit of one of consent norms in the *Children’s Act*. This recommendation is consistent with earlier recommendations for self-consent for adolescents over 12 years to HPV vaccination from Tathia and colleagues^27^ and builds on recommendations from Vawda and colleagues^28^ that the term ‘medical reasons’ is broad enough to include HIV prevention.^28^ We elaborate on earlier recommendations by outlining and using tools of statutory interpretation to justify it.

Following this interpretation, self-consent to medical treatment – understood broadly to include PrEP – is permissible for persons over 12 years only when they have the mental capacity to understand the benefits, risks, social and other implications’ of the proposed treatment.^2,3^

We recommend that service providers should take steps to ensure that those persons who meet the age and capacity requirement for self-consent have access to PrEP.

We also recommend that *policy makers* should ensure that PrEP guidelines are updated to reflect the adolescent consent approach articulated here. Hopefully these efforts will enable at-risk adolescents to access much needed interventions to reduce their risk of HIV.
